# The impact of breast density notification on psychosocial outcomes in racial and ethnic minorities: A systematic review

**DOI:** 10.1016/j.breast.2024.103693

**Published:** 2024-02-22

**Authors:** J.M.J. Isautier, S. Wang, N. Houssami, K. McCaffery, M.E. Brennan, T. Li, B. Nickel

**Affiliations:** a The University of Sydney, Sydney Health Literacy Lab, School of Public Health, Faculty of Medicine and Health, New South Wales Australia; b Wiser Healthcare, School of Public Health, The University of Sydney, New South Wales, Australia; c Faculty of Medicine, The University of Queensland, Brisbane, Australia; d The Daffodil Centre, The University of Sydney, a Joint Venture with Cancer Council NSW, Sydney, New South Wales, Australia; e Westmead Breast Cancer Institute, Westmead Hospital, Sydney, Sydney, Australia; f National School of Medicine, University of Notre Dame Australia, Sydney, Australia

**Keywords:** Breast density, Notification, Impact, Psychological, Awareness, Knowledge, Intention, Ethnic minority, Race

## Abstract

**Background:**

High breast density is an independent risk factor for breast cancer and decreases the sensitivity of mammography. This systematic review synthesizes the evidence on the impact of breast density (BD) information and/or notification on women's psychosocial outcomes among women from racial and ethnic minority groups.

**Methods:**

A systematic search was performed in March 2023, and the articles were identified using CINHAL, Embase, Medline, and PsychInfo databases. The search strategy combined the terms “breast”, “density”, “notification” and synonyms. The authors specifically kept the search terms broad and did not include terms related to race and ethnicity. Full-text articles were reviewed for analysis by race, ethnicity and primary language of participants. Two authors evaluated the eligibility of studies with verification from the study team, extracted and crosschecked data, and assessed the risk of bias.

**Results:**

Of 1784 articles, 32 articles published from 2003 to 2023 were included. Thirty-one studies were conducted in the United States and one in Australia, with 28 quantitative and four qualitative methodologies. The overall results in terms of breast density awareness, knowledge, communication with healthcare professionals, screening intentions and supplemental screening practice were heterogenous across studies. Barriers to understanding BD notifications and intentions/access to supplemental screening among racial and ethnic minorities included socioeconomic factors, language, health literacy and medical mistrust.

**Conclusions:**

A one-size approach to inform women about their BD may further disadvantage racial and ethnic minority women. BD notification and accompanying information should be tailored and translated to ensure readability and understandability by all women.

## Highlights

• Difficulties understanding breast density notifications are common.
• Barriers to breast density notifications include language and health literacy.
• Socioeconomic factors and medical distrust are barriers to accessing supplemental screening.
• A one-size-fits-all approach to breast density notification is not appropriate.
• Culturally and linguistically appropriate breast density notifications are needed.

## 1 Introduction

Breast density (BD) is determined mammographically based on the opacity of breast tissue and reflects the proportion of fibro-glandular relative to fatty tissue [1]. High BD refers to heterogeneously or extremely dense breast tissue (Category C or D density) according to the American College of Radiology Breast Imaging Reporting and Data System classification [2]. While estimates vary, high BD is present in approximately 40% of women of mammography screening age [3]. High breast density can mask breast cancer on mammograms and predispose to an interval cancer diagnosis [4]. Independently, dense breasts also confer a 1.6–2 fold increased risk of breast cancer [5,6].

Largely borne of consumer advocacy by women with dense breasts who developed an interval breast cancer, the United States (US) has a legislated BD notification [7]. This requires women to be informed of their density following a mammography. Connecticut was the first state in the US to introduce BD notification in 2009, with many states following in the subsequent decade [8]. In 2023, the US Federal Drug Administration (FDA) announced a nationwide BD notification mandate with standardised language, which all states must adhere to by September 2024 [1,9]. Other countries are also considering implementing BD notification in national screening programs. For example, BreastScreen Australia, the national breast cancer screening program, does not recommend routine recording of BD [10]. However, BD notification policy varies by state, and is currently implemented in Western Australia and in South Australia [11,12].

The intention of BD notification is to inform women about their BD and empower them to discuss the options to manage it with their health care practitioner (HCP) [7]. This includes using supplemental screening with modalities such as magnetic resonance imaging (MRI), ultrasound or contrast-enhanced mammography. The goal of notification, while ostensibly worthy, brings challenges including lack of consensus on how to guide women and their HCP on best management. There is unclear evidence as to whether supplemental screening offers overall benefit for women with dense breasts in the absence of other risk factors and it may lead to harms such as false positives and overdiagnosis [13]. Indeed, professional groups vary in their recommendations as to what to do about dense breasts with respect to supplemental screening [14]. The emphasis on BD may also reduce the focus on other risk factors for breast cancer such as family history, hormonal and lifestyle factors that may actually more strongly influence breast cancer risk [5]. The BD notification itself is typically written at a high literacy level [15] without extensive testing among diverse populations, and it provides little explanation of BD, the degree of risk, or clear advice, other than to see their HCP, who may themselves feel unprepared about how best to advise women [16].

Consequently, BD notification may cause increased anxiety and confusion, especially among socioeconomically disadvantaged groups such as women with low health literacy, racial and ethnic minorities and including people from culturally and linguistically diverse backgrounds [17]. Moreover, the uptake of supplemental screening, which is not universally covered by insurance in the US nor Medicare in Australia, may be limited to women with financial means and access to these facilities, further exacerbating health inequities which already exist in relation to breast cancer screening and mortality [18,19].

In 2021, our group published systematic reviews on the impact of BD notification on women's cognitive, psychological and behavioural outcomes [20], as well as supplemental screening practice [21]. While race and ethnicity was not the focus of these reviews, we found that several studies demonstrated decreased BD awareness and knowledge in racial and ethnic minorities, who were also less likely to have had supplemental screening, compared to White women [20,21]. The aim of the present systematic review is to focus on the impact of BD notification on racial and ethnic minority groups by synthesising and evaluating the evidence including an updated literature search. Understanding how racial and ethnic minority groups have been impacted by BD notification or BD information, their representation in supplemental screening, and how this compares between racial and ethnic minority groups, is essential to policy planning to address health disparities and inequities both in the US and other countries contemplating BD notification implementation.

## 2 Methods

The review was prospectively registered with PROSPERO (registration number: CRD42023397527) and sought to answer the question, “What is the impact of BD information or BD notification on racial and ethnic minority groups?”. The terms race and ethnicity were chosen to encompass women from different race, ethnic minority, cultural or linguistic backgrounds living in different countries and follows the guidance provided by Flanagin et al. [22] on reporting of race. Women from different racial and ethnic minority populations often face difficulties navigating the health system due to socioeconomic factors, structural, cultural and linguistic barriers as well as the experience of racism. They also may have lower health literacy. The focus on race and ethnicity may elucidate important health disparities and inequities related to BD notifications. The conduct of the review was guided by the Preferred Reporting Items for Systematic Reviews and Meta-Analyses (PRISMA) statement [23].

### 2.1 Search strategy

The review used the same search strategy as our earlier systematic review [20] with broad search terms to capture all relevant articles. Databases MEDLINE, Embase, CINHAL and PsycINFO were searched up to 2 March 2023 for terms “breast”, “density” and “notification” and their variations and synonyms (Supplementary Table 1). Race and ethnicity or related terms were deliberately excluded from the search to broaden the search results. Additional articles identified by collaborators were also included for screening. Search results were uploaded into Endnote (Clarivate, Philadelphia, US) and Covidence (Veritas Health Innovation, Melbourne, Australia; www.covidence.org). After both manual and automatic removal of duplicates in Endnote and Covidence, two researchers (JI, SW) independently screened titles and abstracts for relevance. Subsequently, full-text articles were evaluated for eligibility by predetermined inclusion and exclusion criteria. Any disagreements were moderated by a third researcher (BN).

### 2.2 Eligibility criteria

Eligibility criteria are summarised in Supplementary Table 2. Studies were eligible if they included racial or ethnic status (or a related factor, such as primary or preferred language and country of birth) as a primary study factor or covariate, or if it solely focussed on a racial or ethnic minority group. What constituted racial or ethnic diversity was not predetermined, but defined by authors of the included studies, as this may differ by country or context. Comparison groups, if applicable, could include the general population, or in Western countries, such as the US, non-Hispanic White (henceforth “White”) or Caucasian women. Empirical studies were included if they assessed any impact in relation to BD information or notification, including hypothetical scenarios, on racial and ethnic minorities. Impacts could include cognitive, psychological, or behavioural outcomes or outcomes related to supplemental screening practice. Exclusion criteria included studies of participants under 18 years of age, conference abstracts, protocols, reviews, commentaries or editorials.

### 2.3 Quality assessment and data extraction

Included studies were assessed for quality (risk of bias) using the Joanna Briggs Institute (JBI) critical appraisal tools [24]. JBI tools are designed for use in systematic reviews and cover a range of quantitative and qualitative study designs. Studies are rated as being at low, moderate, or high risk of bias depending on the proportion of “Yes”, “Unclear” or “No” answers to the checklist questions, with low risk of bias indicating high quality (majority “Yes”), and high risk of bias indicating low quality (majority “Unclear” or “No”). Quality was independently assessed by two researchers (JI, SW), with disagreement moderated by a third (BN). Studies were not excluded based on risk of bias.

Data were extracted into an Excel template modified from our previous systematic review [20], covering study characteristics and outcomes relevant to racial and ethnic minority groups. Race and ethnicity was extracted as described by the study authors for each study. Studies were divided so that an equal number were extracted by one author (JI or SW), with the other author checking the extracted data of the other to ensure accuracy and completeness. Results were presented as a narrative review due to heterogeneity of study types and outcomes.

## 3 Results

### 3.1 Study characteristics

**Fig. 1:**
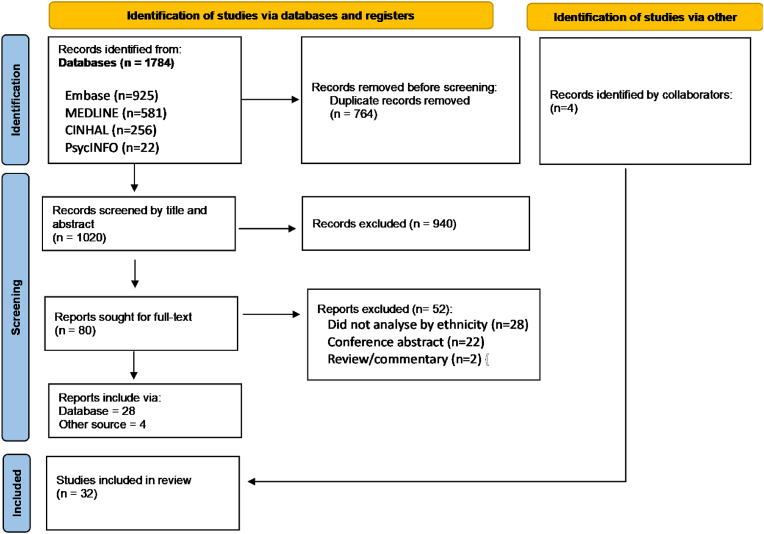
PRISMA flowchart of included studies showing results of searches and review of studies.

Fig. 1 shows the PRISMA flowchart. Of 1784 studies identified by the search, 764 duplicates were removed, and titles and abstracts of 1020 studies were screened. Eighty studies underwent full-text evaluation, along with four studies identified by collaborators, which identified 32 articles for final inclusion in the review.

Table 1 summarises the study characteristics. There were 28 quantitative studies (20 cross-sectional studies including two with a qualitative interview component [17,[25], [26], [27], [28], [29], [30], [31], [32], [33], [34], [35], [36], [37], [38], [39], [40], [41], [42], [43]], five cohort studies including one with a qualitative survey component [[44], [45], [46], [47], [48]], two randomised trials [49,50] and one quasi-experimental study [51], and four qualitative studies (three interviews [[52], [53], [54]] and one focus group [55]). Among quantitative studies, the sample size ranged from 77 to 631,478. One randomised trial compared BD information with information on new imaging technologies [49]. The other trial compared a BD notification letter, a BD notification letter plus a brochure, and a BD notification letter plus a brochure plus a phone call from a Spanish-speaking HCP [50].

**Table 1:** Characteristics of included studies.

Study (author, date)	Country (state)	Pre or post BDN legislation	Study design	Data collection methods	Race and Ethnicity as a main study factor or covariate	Methods used to recruit non-English speakers	Total sample size (N) and race and ethnic minority sample (n, %)	Race and/or Ethnicity N (%)	Personal** or general BD information	Risk of bias^±^
Austin et al., 2021 [44]	US (NY)	Post	Retrospective cohort	Mammography database and face-to-face survey.	Covariate	English and Spanish-speaking survey staff	N = 666 n = 619 (93)	220 (33) Hispanic Mixed/Other 168 (25) Hispanic White 147 (22) Hispanic Black 47 (7) Non-Hispanic White 15 (2) non-Hispanic Mixed/Other	Both	Low
Chau et al., 2017 [45]	US (CA)	Both	Retrospective cohort	Mammography database	Covariate	Nil	N = 631,478* n = 294,135 (47)*	337,343 (53) White 110,246 (17) Asian 93,948 (15) Hispanic 48,115 (8) Black 38,829 (6) Race Unknown 2997 (0) Native American	Personal	Low
Darcey et al., 2021 [46]	Australia (Western Australia)	Post (policy)	Cohort with qualitative component	Online or Telephone survey	Covariate	Nil	N = 6183 n = 407 (7)	5418 (93) Caucasian 338 (6) Missing 217 (4) Asian 190 (3) Other	Personal	Mod
Ezratty et al., 2020 [47]	US (NY)	Post	Retrospective cohort	Mammography database	Main factor	Nil	N = 326 n = 246 (76)	97 (30) Non-Hispanic Black 86 (26) Hispanic 80 (25) Non-Hispanic White 44 (13) Other 19 (6) Asian	Personal	Mod
Gunn et al., 2018 [52]	US (MA)	Post	Qualitative	Telephone interview	Covariate	Nil	N = 29 n = 26 (89)	16 (53) African-American 6 (17) Other/Refused 4 (13) Hispanic 4 (13) Non-Hispanic White	Personal	Mod
Gunn et al., 2019 [53]	US (MA)	Post	Qualitative	Telephone interview	Main factor	Spanish-speaking interviewers	N = 19	19 (100) Hispanic	Personal	Mod
Guterbock et al., 2017 [25]	US (VA)	Post	Cross-sectional	Telephone survey	Covariate	Survey in English or Spanish	N = 1024 n = 273 (27)	751 (77) White 148 (15) Black or African American 22 (2.3) Asian 57 (5.9) Other/multiple race 23 (2) Ashkenazi Jewish 53 (5) Hispanic	General	Low
Kressin et al., 2020 [27]	US (national)	Both	Cross-sectional	Telephone survey	Covariate	Survey in English or Spanish	N = 578 n = Not reported	Not reported	Both	Mod
Kressin et al., 2021 [26]	US (national)	Both	Cross-sectional	Telephone survey	Covariate	Survey in English and Spanish.	N = 2306 n = 1248 (54)	1058 (46) Non-Hispanic White 581 (25) Non-Hispanic Black 338 (14) Hispanic 168 (7) Asian 160 (7) Other	Both	Mod
Kressin et al., 2022a [28]	US (national)	Both	Cross-sectional (qualitative component with brief reference to race)	Telephone survey, interview	Covariate	Survey in English and Spanish.	N = 754 N = 61 (qualitative part) n = 436 (58)	318 (42) Non-Hispanic White 190 (25) Non-Hispanic Black 122 (16) Hispanic 124 (16) Asian/Other	Personal	Mod
Kressin et al., 2022b [29]	US (national)	Both	Cross-sectional (qualitative component with brief reference to race)	Telephone survey, interview	Covariate	Survey in English and Spanish.	N = 2306 n = 1248 (54) N = 61 (qualitative part)	1058 (48) Non-Hispanic White 581 (24) Non-Hispanic Black 338 (15) Hispanic, 168 (9) Asian 160 (5) Other	Both	Mod
Kressin et al., 2023 [17]	US (national)	Both	Cross-sectional	Telephone survey	Main factor	Survey in English and Spanish.	N = 1322 n = 713 (54)	609 (46) Non-Hispanic White 317 (24) Non-Hispanic Black 202 (15) Hispanic 93 (7) Asian 101 (7) Other	Personal	Mod
Kyanko et al., 2020 [30]	US (national)	Both	Cross-sectional	Internet survey	Covariate	Nil	N = 1928 Diversity = 37%	1211 (63) White 248 (13) Black 295 (15) Hispanic 133 (7) Other 42 (2) Mixed	Personal	Mod
Lee Argov et al., 2022 [48]	US (NY)	Post	Cohort	Online, telephone, or mailed surveys	Covariate	English and Spanish-speaking staff and surveys	N = 607 n = 552 (91)	188 (31) Hispanic Mixed/Other 151 (25) Hispanic White 120 (20) Hispanic Black 75 (12) Non-Hispanic Black 56 (9) Non-Hispanic White 17 (3) Non-Hispanic Mixed/Other	Both	Mod
Mahorter et al., 2020 [31]	US (WA)	Post (for specific institution)	Cross-sectional	Mammography database, telephone survey	Covariate	Nil	N = 995 n = 50 (5)	945 (95) White 50 (5) Other	Both	Mod
Manning et al., 2013 [35]	US (MI)	Pre	Cross-sectional	Mailed survey	Main factor	Nil	N = 77 n = 50 (64)	42 (56) Black 26 (34) White 8 (10) Other	Both	High
Manning et al., 2016a [49]	US (MI)	Pre	Randomised trial (2x2 factorial)	Online study	Main factor	Nil	N = 138 n = 67 (49)	67 (49) African-American 71 (51) European American	General	Mod
Manning et al., 2016b [34]	US (MI)	Pre	Cross-sectional	Online survey	Main factor	Nil	N = 295 n = 185 (62)	182 (62) African-American 113 (38) European American	Personal	Mod
Manning et al., 2017 [33]	US (MI)	Post	Cross-sectional	Online survey	Main factor	Nil	N = 452 n = 211 (47)	211 (47) European American 241 (53) African-American	Personal	Low
Manning et al., 2019a [32]	US (MI)	Post	Cross-sectional	Online survey	Main factor	Nil	N = 212 n = 91 (43)	121 (57) European American (43) African-American	Personal	Low
Manning et al., 2019b [51]	US (MI)	Both	Pre-post	Mammography database	Main factor	Nil	N = 3455 n = 2764 (80)	2764 (80) African-American 691 (20) European American	Personal	Mod
Marcus et al., 2022 [55]	US (FL)	Post	Qualitative	Focus group	Covariate	One Spanish-language focus group	N = 25 n = 21 (85)	9 (36) Hispanic/Latina 8 (32) Black 4 (16) White 3 (12) Asian 1 (0) Other	Personal	Mod
Moothathu et al., 2017 [36]	US (CT)	Post	Cross-sectional	Paper survey and mammography database	Covariate	Nil	N = 950 n = 158 (17)	747 (80) Caucasian 98 (10) Black 33 (4) Asian 16 (2) Other 11 (1) Hawaiian 17 (2) Refused to answer 5 (0) Unknown	Personal	Mod
Nguyen et al., 2020 [37]	US (MD)	Post	Cross-sectional	Paper survey	Covariate	Nil	N = 500 n = 175 (35)	325 (65) White 154 (31) Black 13 (3) Asian 8 (2) Other	Personal (hypo-thetical)	Low
O'Neill et al., 2014 [38]	US (DC)	Pre	Cross-sectional	Mailed survey	Covariate	Nil	N = 344 n = 109 (32)	235 (68%) White 84 (24) African American 14 (4) Asian American 6 (2) Native American or Pacific Islander	Both	Low
Pacsi-Sepulveda et al., 2019 [54]	US (NY)	Post	Qualitative	Telephone interview	Main factor	English and Spanish-speaking interviewer	N = 24	24 (100) Hispanic	Personal	Low
Patel et al., 2022 [39]	US (AZ)	Post	Cross-sectional	Online, paper or in person survey	Main factor	Survey in English and Spanish	1479	1479 (100) Latinas	General	Mod
Rhodes et al., 2020 [40]	US (national)	Both	Cross-sectional	Online survey	Covariate	Survey in English and Spanish	N = 1502 n = 353 (24)	1149 (76) White 143 (10) Non-Hispanic Black 98 (7) Hispanic 112 (7) Other or multiple races	General	Low
Richards et al., 2020 [41]	US (national)	Both	Cross-sectional	In-person survey	Covariate	Nil	N = 5701 n = 2052 (36)	3633 (64) Non-Hispanic White 917 (16) Non-Hispanic White 822 (14) Hispanic 329 (5) Non-Hispanic Other	Personal	Mod
Ridgeway et al., 2022 [50]	US (AZ)	Post	Randomised controlled trial	Paper, in person, or phone survey	Main factor	Survey in English and Spanish, Spanish-speaking staff	N = 943	943 (100) Latinas	Both	Mod
Santiago-Rivas et al., 2019 [42]	US (NY)	Post	Cross-sectional	Online and paper survey	Covariate	Survey in English and Spanish	N = 264 n = 219 (83)	127 (48) Black 92 (35) Latina 45 (17) White	Personal (hypo-thetical)	Mod
Yeh et al., 2015 [43]	US (NY)	Post	Cross-sectional	Online survey	Covariate	Nil	N = 184 n = 21 (11)	163 (89) White 16 (9) African-American 9 (5) Hispanic 8 (4) Other	Personal (hypo-thetical)	Low

AZ: Arizona, BD: breast density, BDN: breast density notification, CA: California, CT: Connecticut, DC: District of Columbia, FL: Florida, MA: Massachusetts, MD: Maryland, MI: Michigan, Mod: moderate, NY: New York, SR: systematic review, VA: Virginia, WA: Washington. N = Total sample size, n = race and ethnic minority sample, ^**±**^ Assessed by Joanna Briggs Institute critical appraisal tools [24], *Represents post legislation sample, **Personal BD awareness: participants awareness of their personal breast density category.

All but one study [46] were conducted in the US. Among US studies, there were eight national studies [17,[26], [27], [28], [29], [30],^40^,^41^], six from New York [[42], [43], [44],^47^,^48^,^54^], six from Michigan [[32], [33], [34], [35],^49^,^51^], two from Massachusetts [52,53], two from Arizona [39,50] and one from California [45], Virginia [25], Washington [31], Florida [55], District of Columbia [38], Maryland [37] and Connecticut [36] respectively.

Studies were published between 2013 and 2023. Four were conducted prior to the BD notification mandate [34,35,38,49], 18 post-mandate (including the Australian study, conducted in Western Australia which has a BD notification policy but not a mandate) [25,[31], [32], [33],^36^,^37^,^39^,[42], [43], [44],[46], [47], [48],^50^,[52], [53], [54], [55]], eight US-wide studies capturing women residing in both BD notification and non-BD notification mandated states [17,[26], [27], [28], [29], [30],^40^,^41^], and two studying both pre- and post-legislation periods [45,51]. Four assessed general BD information [25,39,40,49], 16 assessed the woman's personal BD notification [17,28,30,[32], [33], [34],^36^,^41^,[45], [46], [47],[51], [52], [53], [54], [55]], three gave hypothetical BD notifications [37,42,43] and nine assessed both general and personal BD information [26,27,29,31,35,38,44,48,50].

In 12 studies, race and ethnicity (or a related factor) was a main study factor [17,[32], [33], [34], [35],^39^,^47^,[49], [50], [51],^53^,^54^], including four which included only Hispanic, Latina or Spanish-speaking women [39,50,53,54], and in 20 studies race and ethnicity was a covariate [[25], [26], [27], [28], [29], [30], [31],[36], [37], [38],[40], [41], [42], [43], [44], [45], [46],^48^,^52^,^55^]. It should be noted that 12 studies reported on Asian communities [17,25,26,28,29,[36], [37], [38],[45], [46], [47],^55^], however these studies were all conducted in the United States except for one study that was conducted in Australia [47]. The proportion of women in the sample who were racial and ethnic minorities ranged from 5 to 100%. Fifteen studies discussed methodologies specific to the recruitment or data collection from linguistically-diverse women [17,[25], [26], [27], [28], [29],^39^,^40^,^42^,^44^,^48^,^50^,[53], [54], [55]], while in the other 17, no specific methodology was mentioned [[30], [31], [32], [33], [34], [35], [36], [37], [38],^41^,^43^,[45], [46], [47],^49^,^51^,^52^].

Ten studies were assessed as high quality (low risk of bias) [17,25,26,29,30,32,33,37,38,40,[43], [44], [45],^54^], while 22 were of lower quality (moderate or high risk of bias) [27,28,31,[34], [35], [36],^39^,^41^,^42^,[46], [47], [48], [49], [50], [51], [52], [53],^55^]. The study findings did not differ between studies which were considered to be at higher risk of bias and those considered to be at a lower risk of bias.

A summary of outcome measures for each study is presented in Table 2, Table 3, Table 4, Table 5, Table 6, Table 7 and summarised narratively below. Outcomes were categorised as follows: 1) BD awareness (general and personal); 2) BD knowledge (assessed and perceived); 3) BD anxiety or concern; 4) communication with HCPs; 5) screening intentions and supplemental screening practice; and 6) BD notification preferences.

**Table 2:** Summary of results for general and personal** BD awareness.

	Outcomes assessed by race/ethnicity	Results^a^	Summary of reported outcomes related to race/ethnicity
Austin et al., 2021 [44]	General BD awareness	Awareness was significantly lower in women who were Spanish-speaking [OR, 0.16; 95% CI 0.09–0.30 vs. English speakers], were foreign-born (OR, 0.31; 95% CI, 0.16–0.58 vs. U.S.-born), and had lower educational attainment (e.g., high school degree or less; OR, 0.14; 95% CI, 0.08–0.26 vs. college or higher degree).	General BD awareness was lower among Spanish-speaking, Black or Hispanic, and foreign-born women, compared with non-ethnically diverse women, irrespective of previous BDN.
Gunn et al., 2018 [52]	Personal BD awareness	No significant associations were found between those who did and did not recall the notification based on age, race, ethnicity, or primary insurance type.	No difference in recall of BD notification by race/ethnicity among women with dense breasts.
Kressin et al., 2020 [27]	General BD awareness	Non-white race/ethnicity were less likely to have heard of BD (*p* < .05).	Black and Hispanic women were less likely to have heard of BD.
Kressin et al., 2021 [26]	General and personal BD awareness	The likelihood of having heard the term “breast density” differed significantly by race or ethnicity (P < 0.001). White (77.2%) and Asian (75.5%) women more likely to have heard compared to black (60.3%) and Hispanic (49.3%) women, and “other” race (70.1%). Black women (OR 0.79, 95%CI 0.63–1, P = 0.048) and Asian women (OR 0.53, 95%CI 0.37–0.75, P < 0.001) significantly less likely to have received personal BD information.	Black and Hispanic women were less likely to have heard of BD. Black and Asian women were less likely to be aware of their personal BD.
Kyanko et al., 2020 [30]	Personal BD awareness	Black (OR 0.62; 95%CI 0.45–0.85) and Hispanic (OR 0.73, 95%CI 0.55–0.96) women less likely than white women to report having increased BD, adjusting for other covariates.	Black and Hispanic women less likely to report having increased BD.
Mahorter et al., 2020 [31]	General BD awareness	White women significantly more likely to have been aware of BD prior to the study compared to non-white women (91.2% vs 80.8% p < .05), however this association was no longer significant after adjusting for other covariates (BSCS breast cancer risk, income, health status, mammography frequency, health literacy, numeracy).	Non-white women less likely to have been aware of BD prior to the study, and less likely to have discussed BD with a HCP, however no associations after adjusting for covariates. No racial differences in BD knowledge.
Manning et al., 2013 [35]	Personal BD awareness	When we restricted the sample to only the Black and White women, results indicated that knowledge of one's own BD was marginally associated with race (χ2(1) = 3.39, p = .07). Black women were less likely to report knowing their own BD (26 observed vs. 22.7 expected), whereas White women were more likely to report knowing their own BD (observed = 12, expected = 8.7).	Black women less likely to know their own BD compared to White women.
Manning et al., 2016b [34]	Personal BD awareness	European American women were more likely report knowing their BD (42% vs 15%, χ2 (1) = 26.34, p < .0001) compared to African American women.	African American women were less likely to know their own BD
Manning et al., 2017 [33]	Personal BD awareness	Most women (59%) reported no prior awareness of BD; however, statistically significantly more European American women reported prior awareness (58% vs 26%, χ2(1) = 48.03, p < 0 0.01).	African American women had less prior BD awareness.
Moothathu et al., 2017 [36]	Personal BD awareness	Caucasian were more aware of having dense breast (93% vs 86%, p = .0035)	Among women with dense breasts, non-Caucasian women were less likely to be aware of their BD.
O'Neill et al., 2014 [38]	General and personal BD awareness	White-women were more likely to have general BD awareness (OR 2.22 (95%CI 1.15–4.30), *P* < 0.05)	Non-White women were less likely to have general BD awareness.
Pacsi-Sepulveda et al., 2019 [54]	Personal BD awareness	Eleven participants acknowledged that they had received some written report informing them that they had dense breasts; to the remaining 13 participants, BDN information was new.	Majority of Hispanic women could not recall receiving a BDN (all should have).
Patel et al., 2022 [39]	General BD awareness	The National Representative cohort was more likely to be aware of BD than the Arizona cohort (32.6% versus 20.7%, respectively, *P* < 0.005). Awareness was positively associated with more education, prior mammography history, and English language.	Latinas from a low-resource setting had lower BD awareness and knowledge than a national sample of Latinas. Awareness differences were explained by education, preferred language and screening history.
Rhodes et al., 2020 [40]	General BD awareness	BD awareness of significantly lower for Hispanic vs White non-Hispanic women (OR 0.25 95%CI 0.16–0.41, *P* < .0001) and for Black vs Hispanic vs white non-Hispanic women (OR 0.55 95%CI 0.35–0.85, *P* < 0.05), and awareness increased with income and education level (P < .001).	General BD awareness was lower for Hispanic and Black women compared to White non-Hispanic women.
Richards et al., 2020 [41]	Personal BD awareness	Model-adjusted risk ratios for notification were lower than the reference group for women who were aged >55 years, were Black or Hispanic, had not had a mammogram in the past year, were born outside the USA, were not a college graduate, or had income <250% of the federal poverty threshold.	Hispanic and Black women (and foreign-born, compared to US-born women) less likely to have received a BDN.
Ridgeway et al., 2022 [50]	General and personal BD awareness	Participants receiving the interpersonal intervention (a letter plus a brochure and telephonic promotora education) were more likely (P < 0.001) to report seeing their BD results in the letter (70.2%) than those receiving usual care (53.1%) or those receiving a letter plus a brochure (55.1%).	Hispanic women who received a letter plus a brochure and telephonic promotora education were more likely to be aware of receiving their breast density.

a Results as reported in the article. BD = breast density, 95% CI = 95% Confidence Interval, OR: odds ratio, ** personal BD awareness: participants' awareness or knowledge of their BD category.

**Table 3:** Summary of results for breast density knowledge and perceived breast cancer risk.

Study (author, date)	Outcomes assessed by race/ethnicity	Results^a^	Summary of reported outcomes related to race/ethnicity
Gunn et al., 2019 [53]	BD knowledge	Women struggled to understand the 3 components of the notification: (1) dense breasts are considered normal; (2) dense breasts increase your future risk for developing breast cancer; and (3) dense breasts reduce the sensitivity of mammography to detect a cancer (masking bias).	Themes among Spanish-speaking women including confusion due to the novelty of BDN and receiving BDN in English, misinterpretation of key messages.
Guterbock et al., 2017 [25]	BD knowledge	African-American women remain somewhat less familiar and knowledgeable than others, even with socioeconomic indicators controlled. However, the coefficients for African-American status are smaller in the multivariate result than in the bivariate result, suggesting that some but not all of the bivariate race effect is associated with education and socioeconomic differences between Blacks and other Virginia women. Ashkenazi women in Virginia are overall only a little below average in their familiarity and knowledge of breast density, but that they are far below the knowledge levels one would predict given their high socioeconomic status.	Black women were less knowledgeable about BD, both before and after adjusting for socioeconomic covariates (although the adjusted association was weaker). Ashkenazi Jewish women were also less knowledgeable after adjusting for covariates.
Kressin et al., 2020 [27]	BD knowledge	When asked does BD mean how breast feel when you touch them, Hispanic women were more likely to answer incorrectly (*p* < 0.05). When asked whether BD makes it more difficult for a mammogram to correctly detect cancer, White and Hispanic women and those with higher incomes, more education, and aged 65+ were less often correct in bivariate analyses; none remained significant in multivariate results.	Hispanic women scored lower on some aspects of knowledge.
Kressin et al., 2021 [26]	BD knowledge	Women were significantly more likely to recognize the increased risk of breast cancer if they were Hispanic compared with non-Hispanic White (OR 1.39; 95% CI.06–1.83; P = 0.018). Women were significantly less likely to recognize breast density's masking effect on mammography if they were Hispanic compared with non-Hispanic White (OR0.57; 95% CI0.42–0.76; P = 0.001)	Hispanic women had variability in BD knowledge (more likely to know some indicators and less likely others).
Kressin et al., 2022a [28]	BD knowledge	Does BD mean breast feel when one touches them? Non-Hispanic white women more likely correct than black or Hispanic women, still significant after controlling for health literacy. Does BD mean what breasts look like on mammogram? No significant differences by race/ethnicity. Does BD mean the amount of fatty vs connective tissue? White women less likely to respond correctly than black women. Quotes from qual interviews demonstrate that women across literacy status and racial/ethnic backgrounds had misunderstandings about the relationship of fatty tissue to breast density. Do dense breasts increase one's risk of breast cancer? No significant differences by race/ethnicity. Quotes show that women from multiple race/ethnic and literacy groups have varying understandings of the concept of cancer risk associated with breast density, which are not completely explained by quantitative results.	Differences in knowledge by race were variable - on some indicators, there was no racial difference, in others, Black and Hispanic women were less likely to be correct, in others, Black women were more likely to be correct. Qualitative interviews showed no patterns by race/ethnicity.
Kressin et al., 2023 [17]	BD knowledge	Asian women were about a third as likely to report feeling informed compared with non-Hispanic White women (OR: 0.37; 95% CI: 0.21–0.66]).	Asian women were less likely to feel informed than White women.
Lee Argov et al., 2022 [48]	Perceived breast cancer risk	Increased short-term uncertainty about breast cancer risk (OR 1.97, 95% CI 1.15–3.39) for women reporting awareness of breast density (vs unaware), whose dominant interview language was Spanish (i.e. effect modification by preferred language), but not for those whose dominant language was English (OR 1.01, 95% CI 0.58–1.75 and OR 0.99, 95% CI 0.54–1.81, respectively).	Spanish-speaking women who were aware of BD (general or personal) were more uncertain about breast cancer risk. There was no difference among English-speaking women.
Mahorter et al., 2020 [31]	BD knowledge	No significant difference in knowledge of BD effect on mammography between White and Non-white women.	No racial differences in BD knowledge.
Manning et al., 2013 [35]	BD knowledge	ANOVA showed marginal omnibus between-race mean differences in BD accuracy (F2, 67 = 2.86, p = .06), and planned Black–White contrasts demonstrated that White women had significantly more accurate BD definitions (M = 2.77, SD = 0.93) than Black women (M = 2.27, SD = 0.96) t67 = 2.07, p < .05, d = 0.53. Importantly, a race-by-education ANOVA among Black and White women yielded no significant race effect or race-by education interaction. There was only a significant main effect of education (F1, 60 = 17.23, p < .01, η2 = 0.22). No racial difference in knowledge of breast density in relation to breast cancer risk.	Black women have lower BD knowledge accuracy, although this was not significant after controlling for education. No racial difference in knowledge of BD breast cancer risk.
Manning et al., 2016b [34]	BD knowledge	European American women had greater BC risk knowledge (2.85; F1,289 = 52.61, p < .001) and BC risk perception (39.74 vs (0.68 vs. 0.54; F1,289¼ 56.93, p < .001), BD knowledge (3.85 vs. 32.26; F1,232 = 5.53, p < .05) compared to African American women. Among African American women, those with dense breasts had lower perception of breast cancer risk than those without dense breasts.	African American women had less BC risk knowledge, BC risk perception and BD knowledge. Among African American women, those with dense breasts had lower perception of breast cancer risk than those without (demonstrating misunderstanding).
Manning et al., 2017 [33]	BD knowledge	African American women had lower BC risk knowledge, lower BD knowledge, greater scores on all group-based medical mistrust (GBMM) subscales, and reported more group-based and personal discrimination (P < .01).	African American women had less breast cancer risk knowledge, and BD knowledge.
Nguyen et al., 2020 [37]	BD knowledge	Race was not an independent predictor of increasing the likelihood of selecting the appropriate associated lifetime breast cancer risk.	No significant difference by race in likelihood of selecting the correct breast cancer risk.
Pacsi-Sepulveda et al., 2019 [54]	BD knowledge and perceived breast cancer risk	When asked to describe their understanding of the density information, several participants used terms such as “abnormal,” “not normal,” “wrong,” or “not right” to describe their interpretation of the information. A few participants stated that having dense breasts may indicate the existence of breast cancer.	Hispanic women were uncertain about the meaning of BD, perceiving it to be abnormal.
Patel et al., 2022 [39]	BD knowledge	Among women aware of BD, the national representative sample cohort had greater understanding of the masking effect of BD (67/8% vs 37%, P = .001) and breast cancer risk (72.2% vs 32.6%, P < 0.0001)	Latinas from a low-resource setting had lower BD awareness and knowledge than a national sample of Latinas.
Rhodes et al., 2020 [40]	BD knowledge	Black women had lower knowledge of BD masking effects (OR 0.48 (95%CI 0.27–0.85), P < 0.05) after adjusting for covariates.	Knowledge of masking effect of BD was lower for Black women than White women.
Ridgeway et al., 2022 [50]	BD knowledge	All groups saw significant (P < 0.001) but nondifferential improvements in their knowledge of BD as a masking and risk factor.	All women had improved knowledge after the BD notification with no difference between groups.

a Results as reported in the article. BD: breast density, OR: odds ratio, 95% CI = 95% Confidence Interval.

**Table 4:** Summary of results relating to BD anxiety, confusion and breast cancer worry.

Study (author, date)	Outcomes assessed by race/ethnicity	Results^a^	Summary of reported outcomes related to race/ethnicity
Gunn et al., 2019 [53]	BD anxiety and confusion	Overall women receiving the letter in both English and Spanish led to initial reactions dominated by fear and anxiety. Many were frustrated that they received the notification in English when Spanish was their primary language and this led to a delay in understanding what it meant and prolonged worry related to what the letter said. Women found the content in the notification and lack of explanation about BD confusing.	Themes among Spanish-speaking women included confusion and anxiety due to the novelty of BDN and receiving BD notification in English
Kressin et al., 2020 [27]	BD anxiety and confusion	Among women receiving BD notification: Black women reported significantly more anxiety (OR 15.28, 95%CI 4.22–55.27, *P* < 0.05) and confusion (OR 6.66 95%CI 1.31–33.92, *P* < 0.05).	Black women who had received a BDN had higher levels of anxiety and confusion.
Kressin et al., 2023 [17]	BD anxiety	Non-Hispanic Black women (19%), Hispanic women (18%), and Asian women (21%) were significantly more likely to report feeling anxious than were non-Hispanic White women (11%) and women of “other” race/ethnicity (8%) (all *p* < 0.05). These differences persisted for non-Hispanic Black and Asian women in multivariable analyses controlling for other sociodemographic variables. Asian women were almost three times as likely to report feeling anxious than non-Hispanic White women ([OR]: 2.99; 95% confidence interval [CI]: 1.58–5.65), and non-Hispanic Black women were almost twice as likely to report feeling anxious than non-Hispanic White women (OR: 1.76; 95% CI: 1.14–2.70).	Black and Asian women were more anxious about their personal BD. Asian women were less likely to feel informed and more likely to feel confused.
Manning et al., 2016b [34]	BD anxiety and breast cancer worry	African American women indicated greater physical (1.49 vs. 1.25; F1,288 = 8.21, p < .01) and social (1.47 vs. 1.28; F1,288 = 4.96, p < .05) dimensions of BD anxiety, and less BC Worry (2.34 vs. 2.72, F1,291 = 10.09, p < .01). MANCOVA for the emotion outcomes indicated no main effects of race, actual BD, or significant interactions on any of the emotion outcomes.	African American women had higher BD anxiety (however latter moderated by covariates).
Manning et al., 2017 [33]	BD anxiety, BC worry and BD confusion	American African women indicated more physical, social and emotional negative emotionality (*p* < 0.01) and greater anxiety (*p* < 0.05) in response to BD notifications; however, there were no racial differences for BC worry or BD confusion. Prior awareness of BD alleviated general confusion more for African American than European American women.	African American women had greater negative emotionality and anxiety in response to BDN (but not worry or confusion), partly attributable to discrimination and socioeconomic disadvantage
Marcus et al., 2022 [55]	BD anxiety and confusion	Subthemes identified a feeling of fear on learning of increased breast density results; a concern about what causes increased breast density and whether it can be reversed.	A Spanish-language focus group theme included distress waiting for verbal BD notification after mammogram.
Moothathu et al., 2017 [36]	BD anxiety	No statistical difference in anxiety related to breast density awareness (42% and 39%).	No racial difference in BD anxiety.
Pacsi-Sepulveda et al., 2019 [54]	BD anxiety, worry and confusion	Many participants expressed negative emotional reactions to BD notification information, including feelings of worry, stress, and anxiety. Participants' explanations revealed that these emotions were linked to a sense of vulnerability invoked by the BD notification emphases of the “possibility of getting cancer” and the danger of missed cancer.	Hispanic women expressed anxiety about developing or missing cancer.

a Results as reported in the article. BD: breast density, OR: odds ratio, 95% CI = 95% Confidence Interval.

**Table 5:** Summary of results about communication with healthcare practitioners.

Study (author, date)	Outcomes assessed by race/ethnicity	Results^a^	Summary of reported outcomes related to race/ethnicity
Gunn et al., 2019 [53]	Communication with HCP	Women took varied actions to seek further information: Women placed great importance on deciphering the meaning of the notification through information seeking - internet, friends, and family as well as medical providers in which women placed the most importance. Most women had already spoken with their doctor or had intentions to do so. Unrealized expectations and preferences for follow-up: Many women interpreted the receipt of a letter to indicated that additional tests or visits were forthcoming. Women expected doctors to initiate follow-up and some women interpreted the lack of action to mean that everything was normal.	Themes among Spanish-speaking women included seeking further information from a variety of sources, and expectations for follow-up not being met.
Kressin et al., 2020 [27]	Communication with HCP	Hispanic women were more likely to have plans to discuss their personal BD with a doctor (OR 13.26, 95%CI 1.25–140.36, *p* < 0.05). Among women receiving a BD notification, there was no racial difference in past discussion about BD information with a doctor.	Hispanic women were more likely to have plans to discuss their personal BD with a doctor, although there was no racial difference for past discussion with a doctor.
Kressin et al., 2021 [26]	Communication with HCP	Asian women compared with non-Hispanic White women (OR = 0.46; 95% CI = 0.32–0.68; P < 0.001) were less likely to have had past discussion with their HCP. Multivariable analysis revealed that women were significantly more likely to report plans for future discussions with a provider if they had incomes <$50,000 versus $100,000 (OR 1.60; 95% CI 1.17–2.20; P 0.004); were non-Hispanic Black (OR 1.40; 95% CI 1.04–1.90; P 0.028), Hispanic (OR 2.13; 95% CI 1.44–3.14; P.001), or Asian (OR 1.73; 95% CI 1.13–2.63; P 0.011) versus non- Hispanic White; or had low health literacy versus high health literacy (OR 1.88; 95% CI 1.36–2.59; P 0.001).	Asian women were less likely to have had a BD discussion a healthcare provider. Black, Hispanic and Asian women were more likely to be planning to discuss their BD with a healthcare provider.
Mahorter et al., 2020 [31]	Communication with HCP	Weak evidence of higher likelihood of white women vs non-white to have discussed personal BD with a provider (60.2% vs 45.5%, p < .1), however non-significant after adjusting for covariates (family history of breast cancer, prior biopsy, menopausal status, mammography frequency, breast cancer worry).	Non-white women less likely to have been aware of BD prior to the study, and less likely to have discussed BD with a HCP, however no associations after adjusting for covariates.
Manning et al., 2016a [49]	Communication with HCP	BD information, in contrast to information about new breast imaging technology, leads to more favourable intentions to discuss BC screening with one's physicians. The effect of information on intentions was mediated more strongly by behavioural attitudes for African American women compared to European American women.	BD information more so than new technology information increased intentions to talk to physicians about screening for both European American and African American women, however for African American women this effect showed greater mediation by attitudes.
Manning et al., 2016b [34]	Communication with HCP	European American women were more likely to report being advised that they had dense breasts whether they had dense breasts (54.5% vs.27.8%; χ2 [1] = 5.81, p < .05) or not (30.3% vs. 17.1%; χ2 [1] = 4.63, p < .05). HCP communication was associated with greater BC risk knowledge and BD knowledge for African American women whereas it was only marginally associated with BD knowledge for European American women. For African American women, HCP communication was generally associated with less anxiety across physical, social and emotional dimensions, whereas there were no similar associations for European American.	African American women were less likely to have been advised about their BD by a HCP compared to European American women. HCP communication was associated with increased knowledge for African American women and reduced BD anxiety for African American women.
Manning et al., 2017 [33]	Communication with HCP	African American women had stronger intentions than European American women to discuss notification with their physicians. Race-based medical mistrust, perceptions of discrimination, and socioeconomic status accounted for between-race differences in women's intentions to discuss the BD notifications with their physicians.	African American women had more favourable attitudes and intentions to discuss BD notification with HCP. Intention was reduced by socioeconomic disadvantage and mistrust, but not by anxiety, and increased by perception of discrimination.
Manning et al., 2019a [32]	Communication with HCP	No main effects of race, nor interaction between race and prior breast density awareness, on self-reported behaviour; 34% of African American women and 27.3% of Caucasian American reported talking to their health care professionals about BD notification.	No effect of race on behaviour (talking to HCP about their BD notification).
Nguyen et al., 2020 [37]	Communication with HCP	Race was not an independent predictor of increasing the likelihood of patient-initiated discussion with a provider.	No significant difference by race in likelihood of selecting the correct breast cancer risk or initiating discussion with a HCP between existing and revised BD notification.
O'Neill et al., 2014 [38]	Communication with HCP	There was no difference in likelihood of talking to a HCP about BD by race.	There was no difference in likelihood of talking to a HCP about BD by race.
Pacsi-Sepulveda et al., 2019 [54]	Communication with HCP	Although participants generally acknowledged their personal responsibility in adhering to breast cancer screening, they stressed a need for guidance and referrals from their providers and felt that consulting with their providers was an important first step. Few participants reported having had such discussions with their providers.	High motivation to consult with HCP in Hispanic women (although few had done so).
Ridgeway et al., 2022 [50]	Communication with HCP	The percentage of women receiving the interpersonal intervention (a letter plus a brochure and telephonic promotora education) who reported speaking with a provider about BD (29.0%) was significantly greater (P < 0.001) than the percentage of usual care (14.7%) or those receiving a letter plus a brochure (15.6%).	Hispanic women receiving the interpersonal intervention (a letter plus a brochure and telephonic promotora education) were more likely to be aware of receiving their BD notification and have spoken to a HCP than usual or those receiving a letter plus a brochure group.

a Results as reported in the article. BD: breast density, BDN: breast density notification, HCP: health care practitioner, OR: odds ratio, 95% CI = 95% Confidence Interval.

**Table 6:** Summary results about screening intentions and supplemental screening practices.

Study (author, date)	Outcomes assessed by race/ethnicity	Results^a^	Summary of reported outcomes related to race
Chau et al., 2017 [45]	Supplemental screening practice	There was a statistically significant increases in MRI rates for Asian and white women. No significant changes for Black, Hispanic or Native American women pre and post legislation. Compared to White women, Asian, Black and Hispanic women had significantly lower odds of having an MRI, adjusted for other covariates including BD (post legislation).	Asian, Black and Hispanic women were less likely to have a supplemental MRI post-legislation, adjusted for other covariates including BD.
Darcey et al., 2021 [46]	Supplemental screening practice	Ultrasound uptake: Ethnicity was not a predictor for having an ultrasound due to their breast density (P = 0.441).	No difference in ultrasound uptake by ethnicity.
Ezratty et al., 2020 [47]	Supplemental screening practice	Non-Hispanic Black and Hispanic women less likely to have supplemental imaging ordered compared to white women (15% and 10% respectively vs 45%, P < 0.00001). After controlling for patient age, ordering physician specialty, insurance, BI-RADS score, breast density, and family history of breast cancer, non-Hispanic black and Hispanic women remained less likely to be ordered supplemental imaging (OR 0.38 [95% CI 0.17–0.85] and OR 0.24 [95% CI 0.10–0.61], respectively, p < .0001).	Black and Hispanic women were less likely to have supplemental imaging ordered within 12 months of a mammogram showing dense breasts, controlling for insurance and ordering physician type.
Kressin et al., 2023 [17]	Screening intentions	Compared with non-Hispanic White women, both non-Hispanic Black women (OR: 2.04; 95% CI: 1.46–2.84) and Asian women (OR: 2.07; 95% CI: 1.22–3.51) were twice as likely to indicate they were more likely to undergo their next mammogram.	Black and Asian women would be more likely to undergo their next mammogram in reaction to knowing their BD.
Lee Argov et al., 2022 [48]	Screening intentions	Increased short-term uncertainty about breast cancer screening choices (OR 1.73, 95%CI 1.01–2.97) for women reporting awareness of breast density (vs unaware), whose dominant interview language was Spanish (i.e. effect modification by preferred language), but not for those whose dominant language was English (OR 1.01, 95% CI 0.58–1.75 and OR 0.99, 95% CI 0.54–1.81, respectively).	Spanish-speaking women who were aware of BD (general or personal) were more uncertain about breast screening choices. There was no difference among English-speaking women.
Manning et al., 2019b [51]	Supplemental screening practice	Results indicated a 5-fold increase (from 0.14% to 0.7% of women) in supplemental screening among screen-negative women after passage of the law, driven in large part by an increase in supplemental screening among African American women. African American women were less likely to be supplementally screened both before and after the notification law, and invariance tests indicated no difference in magnitude of the between-race difference over time. Breast density was more predictive of supplemental screening and had a marginally greater explanatory role in between-race differences in supplemental screening after passage of the law.	Five-fold increase in supplemental screening post-legislation, largely due to increase among African American women. However, African American women less likely to have supplemental screening both pre- and post-legislation, partially accounted for by decreased BD. Post-legislation, there was no difference in supplemental screening by breast cancer risk in African American women, but among European American women, those supplementally screened had lower breast cancer risk.
Moothathu et al., 2017 [36]	Supplemental screening practice	Caucasian more likely to have had prior ultrasound (79% vs 67%, p = .0028) Non-Caucasian women were more likely to have the screening breast ultrasound only because doctor has ordered it (83% vs 73%, p = .012)	Among women with dense breasts, non-Caucasian women were less to have had prior ultrasound screening, and more likely to have had ultrasound screening solely on doctor's advice.
Pacsi-Sepulveda et al., 2019 [54]	Screening intentions	No one reported undergoing supplemental screening tests because of dense breasts.	No one reported undergoing supplemental screening (sample - Hispanic).
Santiago-Rivas et al., 2019 [42]	Screening intentions	No association between control variables (incl race, country of birth) with intentions to complete mammograms in the future or intentions to complete additional breast cancer screening.	No association between race or country of birth with intentions to have future mammograms or supplemental screening based on a hypothetical scenario.
Yeh et al., 2015 [43]	Screening intentions	Minority women significantly more likely to intend to have ultrasound without insurance coverage (*P* = 0.004).	Minority (non-Caucasian) women more likely to intend to have an ultrasound without insurance in response to a hypothetical BD notification.

a Results as reported in the article. BD: breast density, MRI: magnetic resonance imaging, OR: odds ratio, 95% CI = 95% Confidence Interval.

**Table 7:** Summary of results related to Breast Density notification preferences.

Study (author, date)	Outcomes assessed by race/ethnicity	Results^a^	Summary of reported outcomes related to race
Gunn et al., 2019 [53]	BD notification preferences	Many women interpreted the receipt of a letter to indicate that additional tests or visits were forthcoming. More than just a desire for future testing, women expected doctors to initiate follow-up.	Spanish-speaking women expressed a preference for timely and understandable information and verbal communication with a doctor to allow questions to be answered.
Kressin et al., 2022b [29]	BD notification preferences	Preferences for receiving BDN from a provider were higher among Non-Hispanic Black women (85%) than Non-Hispanic White women (80%), and significantly higher among both Non-Hispanic White women and Non-Hispanic Black women compared to Asian women (72%) (Ps < 0.05). Preference for receiving BDN from an online portal was higher among Asian women (18%) compared to all other groups (White women 6%; Black women 7%; Hispanic women 6%; other 8%). Qualitative findings detailed women's desires for obtaining BD information from providers, written information, and visual depictions of BD.	Black women had a greater preference for receiving BD notification from a provider and a less from a letter. Asian women had a greater preference for online BD notification and less from a provider. Qualitative interviews showed language difficulties to favour BD notification letter preference.
Marcus et al., 2022 [55]	BD notification preferences	All the women stated that just receiving a letter stating they had increased BD was insufficient. The wanted to be told about their BD in person at the time of the appointment. If this was not feasible, they wanted a telephone call from a person at the centre who could answer their questions.	Spanish-speaking women expressed a preference for timely and understandable information and verbal communication with a doctor to allow questions to be answered.
Pacsi-Sepulveda et al., 2019 [54]	BD notification preferences	We asked participants to suggest how they prefer to receive information regarding breast density, and who would be the right person to provide this information. Nearly all the participants stated that health care providers are the most appropriate source of information regarding BD, with most suggesting primary care physicians as the most suitable type of provider to educate women about BD. Participants also expressed interest in written educational materials, made available in physician offices, mammography sites, or public health clinics.	Hispanic women were highly motivation to consult with HCP and HCP was seen as most appropriate source of BD information.
Rhodes et al., 2020 [40]	BD notification preferences	White women as compared to nonblack, non-Hispanic “other” race women were more likely to want to know their individual BD after being told that there was no consensus on what women with dense breasts should do (OR 2.78, p = .0007).	White women were more likely to want to know about their individual BD even after knowing that there is no consensus on what to do with dense breasts compared to non-black, non-Hispanic and other women.
Ridgeway et al., 2022 [50]	BD notification preferences	The percentage of women satisfied with how they were informed of their BD was greater in those receiving the interpersonal intervention (a letter plus a brochure and telephonic promotora education) than usual care group (80.1% vs 70.7%).	Satisfaction and subjective understanding of BDN was higher in the interpersonal intervention (a letter plus a brochure and telephonic promotora education) group.

a Results as reported in the article. BD: breast density, HCP: health care practitioner.

### 3.2 BD awareness

Seven studies reported on general BD awareness [27,31,[38], [39], [40],^44^,^53^] nine on personal BD awareness (participants awareness of their personal breast density category) [30,[32], [33], [34], [35], [36],^41^,^52^,^54^] and two reported both outcomes as shown in Table 2 [26,50]. All but two of these studies [35,38] were conducted post-legislation. Among studies that compared racial and ethnic minority groups, general BD awareness was consistently lower among Black and Hispanic women compared to White women [26,27,31,38,40,44]. In some studies, adjusting for sociodemographic and medical covariates, such as income and breast cancer risk factors [31] or previous BD notification [44] moderated this difference, while in other studies this difference persisted after adjusting for potential confounders [40]. Patel et al. [39] found that Latina women from a low-resource setting had less general BD awareness than a national sample, accounted for by education, primary language and prior mammography.

Among nine studies comparing personal BD awareness between different grous [26,30,[32], [33], [34], [35], [36],^41^,^52^], eight reported that racial and ethnic minority women, including Black [30,[32], [33], [34], [35],^41^], Hispanic [30,41], Asian [26] or “non-Caucasian” [36] women, were less likely to be aware of their personal BD compared to White women, although some studies did not account for actual BD as a confounder, which can differ by race [26,30,41]. In a qualitative study interviewing 24 Hispanic women who were sent a BD notification, Pacsi-Sepulveda et al. [54] reported that 13 women could not recall receiving it. Ridgeway et al. [50] conducted a trial of interpersonal care (telephone call from a Spanish-speaking HCP, BD notification letter and brochure) compared to a BD notification letter or letter and brochure, finding that women in the interpersonal care arm were more likely to recall their personal BD.

### 3.3 BD knowledge

Sixteen studies [17,[25], [26], [27], [28],^31^,[33], [34], [35],^37^,^39^,^40^,^48^,^50^,^53^,^54^] explored women's BD knowledge, assessed objectively with knowledge questions [[25], [26], [27], [28],^31^,[33], [34], [35],^39^,^40^,^50^,^53^] and/or evaluating perceived knowledge or confusion including perceived breast cancer risk as shown in Table 3 [17,[33], [34], [35],^37^,^48^,^50^,^54^]. Of note, thirteen studies found a difference in BD-related knowledge items between groups [17,[25], [26], [27], [28],^33^,^34^,^39^,^40^,^48^,^50^,^53^,^54^].

Of nine studies comparing knowledge between racial and ethnic minorities and White women [[25], [26], [27], [28],^31^,[33], [34], [35],^40^], eight found lower knowledge among racial and ethnic minorities in at least one domain [[25], [26], [27], [28],[33], [34], [35],^40^], although between-race differences varied by knowledge questions in the majority of these studies [[26], [27], [28],^35^,^40^]. In some studies, adjusting for sociodemographics such as income and education moderated this difference [25,35], while in others this difference persisted after adjusting for sociodemographics [25,34,40]. Guterbock et al. [25] found that Black women were less knowledgeable than White women, partially explained by lower socioeconomic and education levels, while Ashkenazi Jewish women were less knowledgeable after accounting for higher socioeconomic and education levels, suggesting differences specific to cultural groups.

Nine of 19 women in Gunn et al.‘s [53] qualitative study of Spanish-speaking women received their BD notification in English which delayed women's understanding until a translation of the letter could be obtained. Some women receiving a Spanish letter interpreted density as the physical presence of a “mass” which highlighted the importance of adjusting the translation to maintain fidelity with the message's meaning to reduce the likelihood of misinterpreting key messages contained in the notification. In Ridgeway et al.‘s [50] intervention trial, two knowledge questions were answered correctly by only 15% and 20% of Latina women at baseline, but all groups (interpersonal intervention, BD notification letter, and letter with brochure) improved their assessed knowledge at follow-up.

In relation to perceived risk of breast cancer, Lee Argov et al. [48] reported increased uncertainty about breast cancer risk for Spanish-speaking women aware of BD, but not among English-speaking women. In Pacsi-Sepulveda et al.‘s [54] qualitative study of Hispanic women, themes emerged around dense breasts being abnormal and indicating breast cancer, and confusion around perceived contradictory information that dense breasts were normal yet increased the risk of cancer. In contrast, Manning et al. [34] found that African American women with dense breasts erroneously perceived their breast cancer risk to be lower than those without dense breasts.

### 3.4 BD anxiety and concern

Of the eight studies examining emotional reaction to BD information or BD notification [17,27,33,34,36,[53], [54], [55]], seven reported increased anxiety among Black [17,27,33,34], Hispanic [[53], [54], [55]], and Asian [17] women as seen in Table 4. One study found no difference in anxiety by race [36]. Manning et al. [33,34] found that BD anxiety was partly attributable to other covariates including education, income, and reported discrimination. Among two qualitative studies of Hispanic and Spanish-speaking women, factors increasing anxiety included difficulty understanding the BD notification [53] and apprehension around need for further screening and anticipated barriers [54].

### 3.5 Communication with health care professionals

Summary of the results related to communication with HCPs are displayed in Table 5. Manning et al. published a series of four articles between 2016 and 2019 where communication with HCPs were central outcomes. In 2016, Manning et al. [34] reported that African American women were less likely to have spoken about their BD with a HCP regardless of their BD status compared to European American women, however those who had expressed reduced anxiety and increased knowledge, highlighting the importance of HCP communication. Another 2016 Manning et al. study [49] showed that providing BD information (versus information on new screening technology) increased plans of both African American and European American women to talk to HCPs. In 2017, Manning et al. [33] showed that African American women had more favourable attitudes and intentions towards discussing BD with a HCP compared to European American women, but this was reduced by socioeconomic disadvantage and medical mistrust. A follow-up paper looking at behaviours found that among African American women, intentions did not predict behaviour. Predictors of behaviour among African American women included prior BD awareness and anxiety (more likely to talk to a HCP) and mistrust (less likely). However ultimately the likelihood of talking to a HCP did not differ by groups [32].

An additional nine studies also examined communication with a HCP as an outcome [26,27,31,[36], [37], [38],^50^,^53^,^54^]. Three studies undertaking between-race comparisons found that Asian [26] and non-White “Other” [31] or “non-Caucasian” [36] women were less likely to have previously discussed their BD with a provider, while one study showed that Hispanic women were more likely to have plans to discuss BD [27], and one study found no difference between race [38]. In two qualitative studies of Hispanic and Spanish-speaking women, high importance was attached to speaking with a HCP, however only a minority had actually done so [53,54]. In Ridgeway et al.‘s [50] trial among Latina women, those who received the telephone intervention were more likely to have spoken to a HCP for follow-up than those receiving the letter or brochure.

### 3.6 Screening intentions and supplemental screening practice

Table 6 shows the results related to screening intentions and supplemental screening practices. Five studies of screening intentions [17,42,43,48,54] found either no race effect [42], or increased intention to undertake future mammography [17,54] or supplemental imaging [43,54] in the presence of BD information, among racial and ethnic minority women. However, while Hispanic women interviewed by Pasci-Sepulveda et al. [54] expressed high motivation to undergo supplemental screening, none had actually done so. Furthermore, increased uncertainty about screening choices was reported by Lee Argov et al. [48] among Spanish-speaking women aware of BD, but not among English-speaking women.

Five studies reported the effect of BD notification legislation or policy on supplementary screening practice by race and ethnicity [36,[45], [46], [47],^51^] of which four showed that racial and ethnic minorities were less likely to have had supplemental screening than White women [36,45,47,51]. Ezratty et al. [47] found that Black and Hispanic women were less likely to have had supplementary imaging ordered, and Chau et al. [45] demonstrated that Asian, Black and Hispanic women were less likely to have had MRI, both studies controlling for differences in actual BD. Moothathu et al. [36] reported less supplemental imaging in non-Caucasian women compared to Caucasian women but did not adjust for BD. Manning et al. [51] reported a five-fold increase in post-law compared to pre-law supplemental screening mainly due to increased screening in African American women. Darcey et al. [46] found no difference by race and ethnicity, however this was limited by only 7% of the sample being racial and ethnic minorities.

### 3.7 BD notification preferences

Six studies examined culturally and linguistically diverse women's BD notification preferences and preferences varied between groups as shown in Table 7 [29,40,50,[53], [54], [55]]. In qualitative studies of Hispanic and Spanish-speaking women [53,54], verbal BD notification communication with a HCP was preferred due to the ability to ask questions, although additional written information was also favoured, with the latter raised as beneficial due to more time to look up unfamiliar words [29,54]. In Ridgeway et al.‘s [50] trial, satisfaction was higher in Latina women receiving the telephone intervention than BD notification letter alone, but not compared to letter and brochure, despite some women stating they had not read the brochure. Contrastingly, increased anxiety while waiting to receive a verbally-delivered BD notification was discussed during a Spanish-language focus group [55]. Kressin et al. [29] showed that preferences varied by race, with Black and White women favouring BD notification from a HCP, and online BD notification favoured by Asian women.

## 4 Discussion

The literature base examining the impact of BD notification on racial and ethnic minorities encompasses a variety of quantitative and qualitative studies. This review synthesizes these studies by a range of outcomes relating to the intended (and unintended) impacts of BD notification - awareness, knowledge, anxiety, communication with HCPs, supplemental screening intentions and practice, and preferred notification methods. While there is variability in the results, there are some consistent findings for between-race and ethnicity experiences of BD notification.

General BD awareness is consistently lower in racial and ethnic minorities, some of which is accounted for by confounders, especially socioeconomic disadvantage among Black and Hispanic women in the US [31,39,44]. Personal BD awareness was also consistently lower, although how much of this effect was moderated by differences in BD among women of different racial backgrounds is unclear [26,30,41]. Knowledge was also lower among racial and ethnic minorities across the majority of studies in at least one domain encompassing the meaning or implications of dense breasts. This again was partially explained by socioeconomic differences, although language barriers, accuracy of translations, and access to reliable information were additional factors reported in several studies [25,35,53]. One study showed a lower level of BD knowledge in women of Ashkenazi background despite their relatively high socioeconomic status [25]. Comparatively lower awareness and knowledge highlights the need to reach different demographic groups with culturally and linguistically appropriate BD notifications and educational resources, and to ensure patient language preferences are accurately recorded and acted upon in medical settings. Both verbally communicated BD notification and a written reference in combination were favoured in studies of BD notification preferences. [29,53,54] Moreover, as the current FDA-mandated notification does not define what dense breasts are, and studies have shown that many women do not have even this knowledge [27,50,53], consideration should be given to further explanation within the BD notification itself as subsequent decision-making hinges on this understanding.

Difficulty understanding BD information in some studies translated to increasing confusion and misunderstanding, which may lead to increased anxiety, and even, believing that dense breasts indicate cancer [54]. Several studies found increased BD anxiety among Black, Hispanic and Asian women compared to White women [17,27,33,34]. Additional factors increasing anxiety included concerns about accessing further care and healthcare discrimination [33,54]. Anxiety also acted as a mediator to encourage HCP follow-up [32,33]. Studies among Hispanic women showed a strong intentions on communicating with HCPs, however due to personal or structural barriers, intentions did not always lead to behaviour, and only a minority had actually followed-through to see their HCP [53,54]. African American women also had intentions to discuss BD with a HCP [33]. Barriers to communicating with HCPs among African American women included socioeconomic status and medical mistrust [32,33]. Among immigrant women in the US, barriers may additionally include language and cultural differences around the acceptability of discussing cancer and breast health [39]. Importantly, women who had spoken to a HCP about their BD reported greater knowledge and reduced anxiety [34,50], emphasising the need for culturally appropriate follow-up care after BD notification. However, women appear to be reassured after speaking to a HCP despite many HCPs feeling unprepared for these discussions. Attention should be given to upskilling HCPs, not only in the management of women with dense breasts, which is an area known to be problematic among non-specialists [16], but also how to deliver this care in a culturally sensitive manner.

HCPs may refer women with dense breasts for supplemental screening. Racial and ethnic minority groups expressed similar or increased motivation to undergo supplemental screening as White women [17,42,43], however data from practice, both before and after BD notification legislation, shows that racial and ethnic minorities are less likely to have supplemental screening [45,47]. Barriers to supplemental screening may be similar to those affecting communication with HCPs, since the latter is an intermediary step, and non-Caucasian women were more likely to rely only on doctor's advice in deciding on supplemental screening [36]. Such barriers include socioeconomic factors (cost, insurance, transport), health literacy and language barriers, medical mistrust and actual or perceived discrimination [32,33,47]. The implications of reduced access to HCPs and supplemental screening, despite high interest and motivation, is concerning for deepening existing inequities in relation to breast cancer screening and treatment for racial and ethnic minorities [18,19,56].

The findings from this systematic review highlight that a one-size-fits-all approach to inform women about their BD may further disadvantage racial and ethnic minorities and create a greater burden in this population. It is concerning that effective in September 2024, the FDA requires that specific language that cannot be altered be implemented in mammography result letters without consideration of the need for translation and cultural adaption [1,9]. It is imperative that the notification language be evaluated to ensure readability and understandability by women of all backgrounds and cultures. Further to this, there is an urgent need and responsibility to translate BD notification information in multiple languages and write the information in low literacy levels. This will ensure notifications can be adequately understood by all women.

This review has both strengths and limitations. This is the first systematic review to examine the impact of BD notification on racial and ethnic minorities. To ensure finding all relevant evidence, we did not restrict our search terms by reference to race/ethnicity, hence we screened a broad range of studies and included studies with ethnicity (and related variables) ranging from a main study factor to a covariate. However, due to the range of outcomes reported, and a range of ethnic groups and ways in which they were defined, we were unable to perform a meta-analysis and results are summarised narratively. The heterogeneity of how ethnicity was defined and recorded also increased the complexity of comparing between studies - for instance, “Black" women could not necessarily be conflated with “African American", nor “Hispanic" with “Latina". Some studies gave broad groupings, such as “Other" to include a range of ethnicities or simply “non-Caucasian". For example, the term “Asian” is a broad category that include numerous countries of origin and regions and may also mask meaningful differences between Asian ethnic subgroups. Many of the studies, especially where race and ethnicity is analysed as a covariate, explicitly included English-speaking as an eligibility criterion for recruitment, resulting in selection bias and marginalisation of linguistically diverse women in this research. Other studies gave no mention of methodology related to language or racial and ethnic diversity at all, likely resulting in selection bias by default.

Difficulty reaching racial and ethnic minority groups due to language, cultural and socioeconomic barriers is a recognised challenge in clinical research [57]. This may have flow-on effects to the external validity of our review. Of note, all but one of the studies was conducted in the United States, and thus the ethnic groups described in the studies have particular relevance to the US context, perhaps with less relevance for other countries with different populations and health systems. Therefore, more research in different settings with different ethnic/racial groups which are well described in terms of their cultural, migration and socioeconomic background are warranted. Moreover, only two randomised trials (assessing different types and content of density-related information) were included in this review, with the remainder of the quantitative studies predominantly cross-sectional in design (predominantly post-implementation or pre-post implementation of notification), limiting ability to draw causal inferences. Notably, there were no randomised trials assessing notification vs no notification of populations that included racial and ethnic minorities.

As the FDA mandate is progressively implemented, almost half of women undergoing mammography in the US will receive a BD notification indicating they have dense breasts [3]. This is likely to increase the demand for discussions with HCP and for supplemental screening, and may increase anxiety and other unintended effects. These are also crucial considerations for other countries, such as Australia, that are contemplating whether to introduce BD notification policies in women potentially never exposed to the concept of BD. This systematic review shows inequities in the impact of BD notification on racial and ethnic minorities. To ensure racial and ethnic minorities benefit equally from BD notification, it is crucial for policy-makers and researchers to focus on targets to reduce inequities. This review highlights that such targets should include a focus on improving awareness and knowledge of BD among racial and ethnic minorities through culturally and linguistically appropriate BD notification and multimodal resources as well as professional development for HCPs. Importantly, in addition to individual patient and provider factors, attention should be placed on mitigating structural barriers, by reorienting health services such as through the provision of staff and resources in languages other than English and ensuring access to supplemental screening, if indicated, is not denied based on socioeconomic means. Building on the evidence-base to support the implementation of BD notification, particularly the inclusion of racial and ethnic minorities in high quality clinical trials both within and outside of the US, is a research imperative.

## Funding

This work was supported by a NHMRC Emerging Leader Research Fellowship (1194108) awarded to BN, and co-supported by a 10.13039/501100001026National Breast Cancer Foundation (NBCF Australia) Chair in Breast Cancer Prevention grant (EC-21-001) awarded to NH. KM is supported by a NHMRC Investigator (Leader) Fellowship (2016719). NH is supported by a NHMRC Investigator (Leader) Fellowship (1194410). TL is supported by a 10.13039/501100001171Cancer Institute NSW Early Career Fellowship (grant #2022/ECF1420). The funders had no role in the planning, writing or publication of the work. The funders of the study had no role in study design, data collection, data analysis, data interpretation, or writing of the manuscript.

## CRediT authorship contribution statement

**J.M.J. Isautier:** Conceptualization, Formal analysis, Methodology, Writing – original draft. **S. Wang:** Conceptualization, Formal analysis, Writing – original draft, Methodology. **N. Houssami:** Supervision, Writing – review & editing. **K. McCaffery:** Conceptualization, Supervision. **M.E. Brennan:** Writing – review & editing. **T. Li:** Writing – review & editing. **B. Nickel:** Conceptualization, Formal analysis, Methodology, Supervision, Writing – review & editing.

## Declaration of competing interest

The authors have no conflicts of interest to declare.
