# Supplementary material for: The impact of breast density notification on psychosocial outcomes in racial and ethnic minorities: A systematic review
Source: Breast. 2024 Feb 22;74:103693. doi: 10.1016/j.breast.2024.103693 (PMC10918326; doi:10.1016/j.breast.2024.103693)
Supplement: Multimedia component 1 [file mmc1.docx]

**Supplementary Table 1**: Search strategy (modified from Nickel et al., 2021^20^)

| **MEDLINE** | **Embase** | **PsycINFO** | **CINHAL** |
| --- | --- | --- | --- |
| 1. breast*.mp  2. mammogr*.mp  3. 1 or 2  4. inform*.mp  5. notif*.mp  6. communic*.mp  7. legislat*.mp  8. advise*.mp  9. told.mp  10. 4 or 5 or 6 or 7 or 8 or 9  11. dens*.ti  12. 3 and 10 and 11 | 1. breast*.mp  2. mammogr*.mp  3. 1 or 2  4. inform*.mp  5. notif*.mp  6. communic*.mp  7. legislat*.mp  8. advise*.mp  9. told.mp  10. 4 or 5 or 6 or 7 or 8 or 9  11. dens*.ti  12. 3 and 10 and 11 | 1. breast*.mp  2. mammogr*.mp  3. 1 or 2  4. inform*.mp  5. notif*.mp  6. communic*.mp  7. legislat*.mp  8. advise*.mp  9. told.mp  10. 4 or 5 or 6 or 7 or 8 or 9  11. dens*.ti  12. 3 and 10 and 11 | S1 AB breast* OR AB mammogr*  S2 AB inform* OR AB notif* OR AB communic* OR AB legislat* OR AB advise* OR AB told  S3 TI dens*  S4 SI AND S2 AND S3 |
