# Supplementary material for: The impact of breast density notification on psychosocial outcomes in racial and ethnic minorities: A systematic review
Source: Breast. 2024 Feb 22;74:103693. doi: 10.1016/j.breast.2024.103693 (PMC10918326; doi:10.1016/j.breast.2024.103693)
Supplement: Multimedia component 2 [file mmc2.docx]

**Supplementary Table 2**: Eligibility criteria for included studies (modified from Nickel et al., 2021^20^)

| **Criteria** | **Inclusion** | **Exclusion** |
| --- | --- | --- |
| Types of studies | Empirical studies (quantitative and qualitative e.g. surveys, interviews or focus groups) that consider the impact or effect of breast density information / notification / communication / legislation on women; retrospective database studies. | Protocols, review papers, editorials, commentary / discussion papers, conference abstracts / proceedings. |
| Population / types of participants | Racial or ethnic minorities - Women (patients or community members) aged 18 years and above with or without dense breast receiving breast density information (including sub-group analysis). | Women < 18 years of age. |
| Types of setting | Any type of medical or community setting (including hypothetical scenarios given to women). |  |
| Study factor (intervention) | Breast density information and / or notification. |  |
| Outcome factor | Cognitive, psychological and behavioural impact / effect e.g. knowledge, awareness, anxiety, intention.  Supplemental screening or healthcare utilisation pre / post legislation using retrospective data. |  |
